# Effect of Different Drying Techniques on Antioxidant Capacity of Two Romanian Red Grape Cultivars

**Published:** 2019-07

**Authors:** Oprica LACRAMIOARA, Verdes ANDREEA, Poroch VLADIMIR, Creanga DORINA, Grigore MARIUS-NICUSOR

**Affiliations:** 1. Department of Biology, Alexandru Ioan Cuza University, Iasi, Romania; 2. Department of Physics, Alexandru Ioan Cuza University, Iasi, Romania; 3. Faculty of Medicine, Grigore T. Popa University of Medicine and Pharmacy, Iasi, Romania

## Dear Editor-in-Chief

Daily consumption of foods with a high content of antioxidant substances is delaying aging. It protects us from diseases, including cancer. Many recent researches are focused on the relationship between fruit and vegetable intake and health (1–4). Lowering aging and stress oxidative risk associated with diseases may be obtained by a diet rich in fruit and vegetables (5).

The regular consumption of grape may exert health benefits that are mainly attributed to some of their bioactive phytochemicals. Moreover, grapes are known to be highly effective against cardiovascular diseases because of significant amounts of phenolic compound (stilbene, flavonoids, proanthocyanidins, and anthocyanins) (6).

In order to reduce microbial activity and deterioration, and to extend the shelf life of the food products several dehydration methods are available. Drying may affect quality or quantity of phytochemicals (7). Moreover, since fresh grapes are highly perishable, it is recommended to use a dehydration process as an alternative to preserve most of their beneficial properties. By measuring the antioxidant activity/capacity levels of fruits and vegetables, or generally, of foods is being watched the ability to detoxify free radicals, including reactive oxygen and nitrogen species, thus preventing damages to main macromolecules and cell structures.

In this context, the aim of this study was to assess and compare the effect of two drying methods (oven drying and freeze-drying) on antioxidant activity (according to DPPH radical-scavenging capacity) of two red grape cultivars (Cabernet Sauvignon and Merlot) in different parts (seed, skin – epicarp, and pulp - mesocarp); the grapes were collected from two experimental fields (Oct 2015 vintage): Bucium and Copou (North East of Romania).

The fully ripened and matured grapes fruits components were frozen for 24 h (at −80 °C), homogenized with food processor and put into lyophilizer. The trial lasted from 12 to 24 (48 h), under conditions of low temperature and high vacuum. The oven dried grape cultivars components were obtained using gravimetric method by evaporation at mild temperature (105 °C) until they reached a constant weight (8).

Freeze-dried seeds of Cabernet Sauvignon and Merlot grape showed relatively equal values of % DPPH inhibition in both cultivars and sampling locations (Table 1). On the other hand, oven-dried seeds of the two-different grape extracts have approximately the same values in Copou location; lower values were recorded in the case of grape cultivars from Bucium – with large difference in the case of Sauvignon.

The antioxidant activity in the pulp is shown approximately similar values in freeze-dried samples, for both cultivars and locations.

The oven-dried pulp of investigated grape cultivars has a higher antioxidant activity as compared with correspondent freeze-dried samples; in this respect, grapes sampled from Bucium registered increased values than those from Copou.

In the case of the skin, in both freeze-dried and oven-dried extracts, the values were higher in those obtained from Bucium grapes. However, freeze-dried samples have a higher antioxidant than in oven-dried variants.

**Table 1:** Antioxidant activity in freeze-dried and dried components of Cabernet Sauvignon and Merlot grapes cultivar

***Species/Sampling area***	***Freeze-dried grape extracts Mean ± SD***			***Oven-dried grape extracts Mean ± SD***		
	***Seeds***	***Pulp***	***Skin***	***Seeds***	***Pulp***	***Skin***
Cabernet Sauvignon/COPOU	93.88 ±0.04	38.88 ±2.86	85.16 ±1.30	93.66±0.09	49.54±0.38	70.34±2.80
Cabernet Sauvignon/BUCIUM	93.98 ±0.03	35.99 ±0.88	86.77 ±0.94	68.05±5.47	54.94±5.57	81.22±1.56
Merlot/COPOU	93.38 ±0.30	38.56 ±1.15	81.63 ±1.99	94.34±0.22	47.44±1.85	72.75±1.19
Merlot/BUCIUM	94.25 ±0.31	35.83 ±0.44	88.82 ±0.28	92.98±0.04	66.75±1.70	84.82±0.21

By comparing the two drying methods (oven dried and freeze-dried), antioxidant activity was relatively similar in seeds extracts of both cultivars, except the Cabernet Sauvignon (Bucium), where it was lower. Furthermore, the oven-dried pulp extracts have a significantly higher antioxidant activity compared to freeze-dried extracts. On the contrary, the antioxidant activity of the oven-dried skin extracts was slightly lower than that of the freeze-dried extracts.

The antioxidant activity of fruit components of grape cultivars (Cabernet Sauvignon and Merlot) subjected to different drying techniques in view of dehydration, varies depending on the grape part (seed, skin, and pulp), collecting areas and analyzed cultivars.
